# Estrogen Receptor Alpha Is Expressed in Mesenteric Mesothelial Cells and Is Internalized in Caveolae upon Freund's Adjuvant Treatment

**DOI:** 10.1371/journal.pone.0079508

**Published:** 2013-11-14

**Authors:** Petra Balogh, Arnold Szabó, Sándor Katz, István Likó, Attila Patócs, Anna L.Kiss

**Affiliations:** 1 Department of Human Morphology and Developmental Biology, Semmelweis University, Budapest, Hungary; 2 Pharmacology and Drug Safety Research, R. Gedeon Plc, Hungary; 3 HSA-SE Lendület Hereditary Endocrine Tumors Research Group, Budapest, Hungary; Rush University Medical Center, United States of America

## Abstract

Transformation of epithelial cells into connective tissue cells (epithelial-mesenchymal transition, EMT) is a complex mechanism involved in tumor metastasis, and in normal embryogenesis, while type II EMT is mainly associated with inflammatory events and tissue regenaration.

In this study we examined type II EMT at the ultrastructural and molecular level during the inflammatory process induced by Freund's adjuvant treatment in rat mesenteric mesothelial cells. We found that upon the inflammatory stimulus mesothelial cells lost contact with the basal lamina and with each other, and were transformed into spindle-shaped cells. These morphological changes were accompanied by release of interleukins IL-1alpha, -1beta and IL-6 and by secretion of transforming growth factor beta (TGF-β) into the peritoneal cavity. Mesothelial cells also expressed estrogen receptor alpha (ER-α) as shown by immunolabeling at the light and electron microscopical levels, as well as by quantitative RT-PCR. The mRNA level of ER-α showed an inverse correlation with the secretion of TGF-β. At the cellular and subcellular levels ER-α was colocalized with the coat protein caveolin-1 and was found in the plasma membrane of mesothelial cells, in caveolae close to multivesicular bodies (MVBs) or in the membrane of these organelles, suggesting that ER-α is internalized via caveola-mediated endocytosis during inflammation. We found asymmetric, thickened, electron dense areas on the limiting membrane of MVBs (MVB plaques) indicating that these sites may serve as platforms for collecting and organizing regulatory proteins. Our morphological observations and biochemical data can contribute to form a potential model whereby ER-α and its caveola-mediated endocytosis might play role in TGF-β induced type II EMT *in vivo*.

## Introduction

Epithelial-mesenchymal transition (EMT) is a biological process that allows a polarized epithelial cell to undergo several biochemical/morphological changes to gain a mesenchymal phenotype [1]. The process was first described by Elisabeth Hay who also depicted the basic differences of these cellular actions during embryogenesis and tumorigenesis [2]. Since then three subtypes of EMTs have been distinguished with different functional consequences. Besides epithelial-mesenchymal transition during embryogenesis (type I) and tumorigenesis (type III), type II EMT is associated with wound healing, tissue regeneration and organ fibrosis [3], [4]. It has been demonstrated that upon inflammation many cells (monocytes/macrophages, fibroblasts) can trigger type II EMT through secretion of growth factors such as transforming growth factor-beta (TGF-β) or epidermal growth factor (EGF). Most prominent among these cells are the macrophages and activated resident fibroblasts that accumulate at the site of injury and release these growth factors [1], [2], [5]. TGF-β was first described to induce EMT via Smad 2/3-dependent pathway and meanwhile it became evident that the cellular actions of the cytokine can be modulated and defined by other Smad-independent signaling pathways like the MAP kinase pathways [6]]–[[8].

Recently, estrogen receptor alpha (ER-α) has been suggested as another player in the molecular mechanism of EMT [9]]–[[11]. For long, estrogen receptors (ER-α, ER-β) have been considered exclusively as transcription factors acting inside the nucleus [12], [13]. However, the discovery of its membrane-associated form and the ER–mediated transcription in the absence of its ligand generally changed this concept [14], [15]. The theory of a hormon-independent ER-α activation that can serve as a mechanism to amplify growth factor pathways [16] has also been accepted by now. It has been proved that a small percentage of ERs (5–10%) reside in the cell membrane and can elicit both genomic and non-genomic responses by activating multiple protein kinase cascades that include MAPK, protein kinase C, Src kinase and PI3K [17]]–[[23].

ER-α and TGF-β have opposing roles in cell proliferation and differentiation of epithelial cells. Their regulatory pathways intersect and ER-α blocks the TGF-β pathway at different cellular levels inside the nucleus as well as in the cytoplasm and plasma membrane. Both transcription factors have a prominent role in maintaining a controlled signaling that is essential for cell and tissue homeostasis and both act in a cell-specific and context-dependent manner [24]–[25].

In our previously published papers we showed that mesothelial cells that form a continuous squamous epithelial layer on the two sides of the mesentery undergo epithelial-mesenchymal transition (EMT) upon Freund's adjuvant treatment [26]. It has also been proved that upon inflammatory stimuli mesothelial cells can assume a macrophage character demonstrated by the expression of the macrophage marker, ED1 [27] and they can serve as a source of activated macrophages during inflammatory events [28]. Besides this finding, it is also known that macrophages express ER-α [29], [30]. These data together led us to examine whether mesothelial cells express ER-α and if so, what is the subcellular distribution of the receptor. We examined the morphological and biochemical changes of mesothelial cells under inflammatory stimuli *in vivo*. This study reports for the first time that ER-α is expressed by the rat mesenteric mesothelial cells and an inverse correlation between the level of ER-α expression and the peritoneal secretion of TGF-β can be observed in response to Freund's adjuvant treatment. The present results show that ER-α is associated with caveolin-1 protein and the receptor is presumably internalized via caveola-mediated endocytosis. Our morphological observations and biochemical data together indicate that ER-α and its caveola-mediated endocytosis may play role in TGF-β induced type II epithelial-mesenchymal transition.

## Materials and Methods

### Ethics Statement

All rat experiments were carried out in accordance with the recommendations in the Guide for the use of Adjuvants in Research of the National Institutes of Health and approved by the Institutional Animal Care and Use Committee of the University of Massachusetts Amherst [31] and Semmelweis University's Institutional Animal Care and Use Comittee. All efforts were made to minimize suffering.

### Material and Treatment

Rat mesentery (peritoneum of small intestine) was isolated from control and Freund's adjuvant (Sigma, Steinheim, Germany) treated Sprague-Dawley male and female rats (200–400 g). 1 ml complete Freund's adjuvant was injected into the peritoneal cavity. One day (D1), 2 days (D2), 3 days (D3) and 5 days (D5) following intraperitoneal injections the animals were sacrificed by decapitation and mesentery samples were removed.

### Collection of peritoneal fluid, isolation of mesothelial cells

To determine the secretion level of TGF-β, peritoneal fluid (PF) samples were collected from both male and female animals by washing the peritoneal cavity with 2 ml phosphate-buffered saline (PBS). The PF samples were centrifuged with 1000 rpm, for 10 min at 4°C and the supernatants were stored at −20°C until use for immunoblotting.

For isolating mesothelial cells, mesentery was removed and placed into 0,2% collagenase diluted DMEM tissue culture medium (Sigma) for 45–50 minutes at 37°C. The solid remnants (adipose tissue, connective tissue) were removed and samples were washed three times in PBS and centrifuged with 1000 rpm, for 10 min at room temperature. The pellets were then placed in liquid nitrogen for 10 minutes and stored at −80°C until RNA isolation for quantitative RT-PCR.

### RNA isolation and quantitative RT-PCR

Total RNA was extracted with RNeasy tissue mini kit (QIAGEN Inc., Chatsworth, CA). For qualitative and quantitative analysis of RNA preparations, RNA 6000 Nano Chip kits in an Agilent 2100 Bioanalyzer (Agilent Technologies Inc., Santa Clara, CA) was used according to the manufacturer's instructions. Following RNA isolation, RNAs obtained were transcribed into cDNAs using SuperScript™ III First-Strand Synthesis SuperMix (Life Technologies, Carlsbad, CA), according to the manufacturer's instructions. From each samples, 0.5–1 µg of total RNA was converted into cDNA. Real-time PCR was performed using Taqman Universal Master Mix II, No UNG and the 7900HT Real-Time PCR System (Life Technologies, Carlsbad, CA), with the following parameters: 50°C for 2 min and 95°C for 10 min, followed by 40 two-step cycles at 95°C and at 60°C for 1 min. Applied Biosystems pre-designed TaqMan® Gene Expression Assays were used for real-time PCR (Il6 Rn00561420_m1, Il1a Rn00566700_m1, Il1b Rn00580432_m1, Esr1 Rn00664737_m1, GAPD.4352338E, Esr2 Rn00562610_m1, Gpr30 Rn00592091_s1 and B2m Rn00560865_m1). The qRT-PCRs were executed in triplicate using TaqMan probe synthesized by Applied Biosystems). SDS 2.3 (Applied Biosystems, Foster City, CA) and RQ manager 1.2 softwares (Applied Biosystems, Foster City, CA) were applied for calculation of the threshold cycle (Ct) values in each sample.

Expression level was calculated by the ddCt method, and fold changes (FC) were obtained using the formula 2^-^^ddCt^. Computed internal control corresponding to the geometric mean of Ct values of the two houskeeping genes (GAPD and B2m) was used for the ddCt calculation. Gene expression levels at different time points were compared to controls using ANOVA and t-test with a statistical software SPSS version 13 (SPSS Inc., Chicago, IL, USA).

### Antibodies

Primary antibodies were polyclonal anti-caveolin-1 antibody (1∶40 for IEM, Transduction Laboratories) and polyclonal anti estrogen-receptor alpha antibody (H-184): sc-7207 (1∶50-IEM, Santa Cruz Biotechnology, Inc). Biotinylated anti-rabbit IgG (1∶200) was used as a secondary antibody (VECTOR Laboratories Inc, Burlington, Canada). For confocal microscopy, streptavidin Alexa Fluor 488 conjugate and streptavidin Alexa Fluor 555 conjugate (MolecularProbes, Leiden, the Netherlands) were used (1∶200). The nuclei were stained with DAPI (Vectashield DAPI mounting medium, Burlingame, Canada). For immunogold labeling Protein A conjugated to 10- (1: 80) and 15-nm (1∶60) gold particles was manufactured and kindly gifted by the Cell Microscopy Centre, Utrecht, The Netherlands.

### Western blotting

The protein contents of the supernatants from peritoneal fluid samples were determined by the Bradford method [32] and diluted to a concentration of 1 mg/ml. Afterwards the samples were solubilized with reducing Tris-SDS buffer (Tris 0.5 M pH 6.8, 10% glycerol, 2% SDS, 0.00125% bromophenol blue, 0.5% mercapto-ethanol) and heated at 95°C for 4 min. 15 µl per well from the samples were loaded onto an 10% Acrylamide/Bis gel and separated by electrophoresis. After separation, proteins were transferred onto a nitrocellulose membrane (Amersham, GE Healthcare Biosciences, Pittsburgh) in a buffer containing Tris–Glycine pH 8.3, 0.1% SDS and 20% methanol.

Aspecific reactions were blocked at room temperature for 2 h in PBS-Tween (0.5 M PBS, Tween 0.05%) containing 5% skim milk powder. The membranes were then incubated with polyclonal anti-rabbit TGF-β primary antibody (Cell Signaling Technology Inc., Beverly, MA) diluted 1∶1000 at 4°C for overnight. After washing in PBS-Tween the membranes were treated with species-specific peroxydase-conjugated secondary antibody (Amersham, GE Healthcare Biosciences, Pittsburgh) for 1 h at room temperature. The labeled protein bands were visualized by the ECL Plus chemiluminescence method and developed onto high performance chemiluminescence film (Amersham, GE Healthcare Biosciences, Pittsburgh). Relative optical densities were measured using the ImageJ software (U. S. National Institutes of Health, Bethesda, Maryland) and the results of four independent experiments were compared and statistically analyzed. The significance was tested by the ANOVA method and Tukey's HSD test.

### Tissue fixation for light and electron microscopy

Mesentery was isolated from both control and Freund's adjuvant-treated animals. The samples were fixed either in a 1∶1 mixture of 2% glutaraldehyde (GA in 0.2 M cacodylate buffer) and 2% OsO_4_ in distilled water (30 min, on ice) or freshly prepared 4% paraformaldehyde (PFA) in 0.1 M PB (2–4 h, room temperature). The samples were washed in cacodylate buffer and PBS, respectively and the adipose tissue was removed from the mesentery. The GA-Os fixed material was proceeded to electron microscopic embedding, while the PFA-fixed samples were used for immunocytochemistry.

### Electron Microscopy

The GA-Os fixed samples were washed in 0.1 M cacodylate buffer three times, dehydrated with ethanol and stained with 1% uranyl acetate (in 70% ethanol for 1 h, room temperature) prior to araldite embedding. Ultrathin sections were contrasted with uranilacetate and lead citrate. The samples were analyzed in a Hitachi H-7600 (Tokyo, Japan) transmission electron microscope.

### Preparation of semithin and ultrathin cryosections

The PFA-fixed samples were stored in 1% formaldehyde (in 0.1 M PB) at 4°C until further processing. For semithin cryosectioning and immunolabeling, the fixed samples were washed twice in PBS, once in 0.02 M glycin/PBS and infiltrated gradually with gelatine solutions of increasing concentrations (2%–5%–12% in PB) at 37°C for 30 min each. The samples were oriented in liquid gelatin and cut into small blocks. For cryoprotection, the blocks were infiltrated with 2.3 M sucrose at 4°C overnight and afterwards mounted on metal pins, frozen and stored in liquid nitrogen. For preparing semithin and ultrathin cryosections we used Leica Ultracut S ultramicrotome equipped with cryo-attachment (Vienna/Austria). The pickup solution was a 1∶1 mixture of 2.3 M sucrose and 1.8% methylcellulose.

### Immunolabeling for light and electron microscopy

0.5 µm semithin sections mounted on microscopic slides were incubated with 0.02 M glycine in PBS for 15 min and then they were blocked in PBS containing 1% BSA. Primary antibodies were applied in 1% BSA containing buffer in a humidified chamber at 4°C (overnight). Polyclonal anti-caveolin-1 and polyclonal anti estrogen-receptor alpha antibodies were used at the dilution 1∶200 and 1∶100, respectively. After washing with PBS, this was followed by a 1 h incubation with the secondary antibody in a humidified chamber at room temperature. Sections were then rinsed with PBS, stained with DAPI, placed under coverslips and visualized in a Bio-Rad (Ontario, Canada) Radiance 2100 Rainbow confocal microscope.

For cryosectioning and immuno-EM, the fixed tissues were further processed as described [33]. Ultrathin cryosections prepared at −100°C were transferred to copper grids by pickup with a 1∶1 mixture of 2.3 M sucrose in PBS and 1.8% methylcellulose. For immunogold labeling, the grids were incubated first on 2% gelatin/PBS at 37°C, blocked with 0.02 M glycine/PBS and subsequently incubated with primary antibodies followed by protein A-gold. The sections were contrast stained with 2% uranyl acetate/oxalate, pH 7, followed by 0.4% uranyl acetate pH 4 and 1.8% methyl cellulose and dried. The cryosections were analyzed in a Hitachi H-7600 (Tokyo, Japan) transmission electron microscope at 80 kV.

## Results

### Ultrastructural and biochemical evidences of type II EMT in mesothelial cells *in vivo*

**Figure 1:**
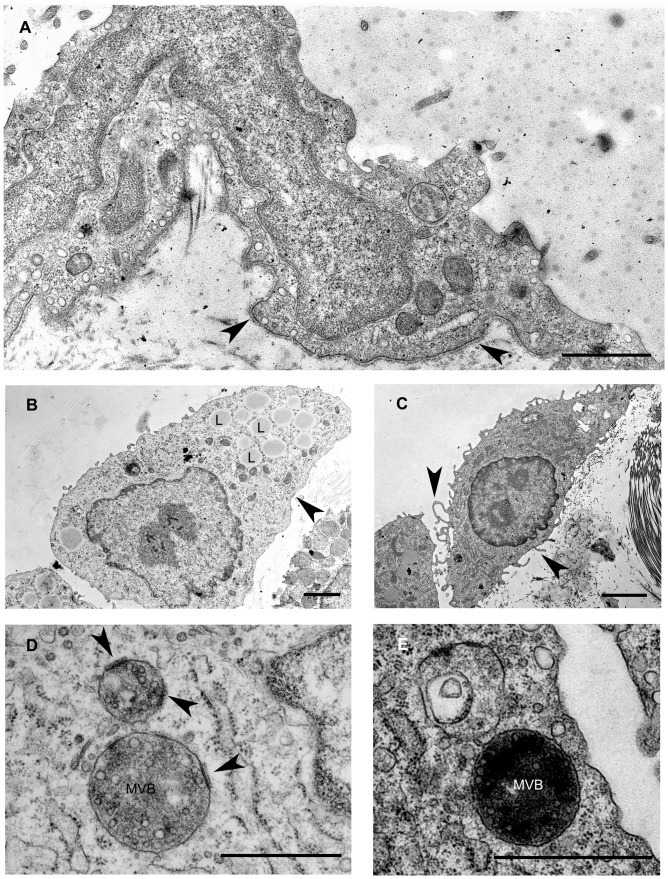
Fine structural aspects of EMT in mesothelial cells upon Freund's adjuvant treatment. (A) Non-treated (control) cells are flat and form a continuous layer on the basal lamina (arrowheads). Caveolae are abundantly present in the control cells seen as small flask shape invaginations of the plasma membrane. (B) Upon inflammation mesothelial cells progressively lose the connection with the underlying connective tissue as the basal lamina becomes discontinuous and disintegrates. The cytoplasm of mesothelial cells contains lipid droplets (L) that are probably identical with internalized Freund's adjuvant oil droplets. (C) On the fifth day of treatment, mesothelial cells assume a spindle-shape morphology with several villar or lamellar processes (arrowheads) on their surface. (D) An increasing number of multivesicular bodies (MVB) appear from D3. Note specific membrane domains of the organelles (arrowheads) showing an increased density and apposition of a coat on the cytosolic side (MVB plaques). (E) The MVB plaques disappear by the later stages of MVB maturation, by D5. Scale bars: (A) 1 µm (B) 2 µm (C) 2.9 µm (D) 500 nm, (E) 1 µm.

In untreated, control animals mesothelial cells form a continuous simple squamous epithelial layer on both sides of the mesentery. As observed in the electron microscope, the cytoplasm of these cells was rather poor in cell organelles and the cells were dominated by the nucleus. A continuous basal membrane separates the mesothelial cell layer from the underlying connective tissue and maintains the integrity of the mesothelial layer. Caveolae (caveolin-containing lipid rafts seen as flask-shaped invaginations of the cell membrane) were abundantly present on the basolateral and apical surfaces of the cells (Fig. 1A).

Our previous light microscopical data revealed that mesothelial cells lose their epithelial character (decreased cytokeratin, E-cadherin expression) upon Freund's adjuvant treatment and obtain a mesenchymal phenotype by expressing vimentin [26]. The inflammatory stimuli changed remarkably the ultrastructure of mesothelial cells as well. These changes culminated on the second to third day (D2/D3) after treatment. From the fifth day (D5) on the tissue started to recover and the repairment was morphologically accomplished by the eleventh day (D11). Characteristic changes were the disintegration of basal membrane and transformation of the squamous mesothelial cells into individual cuboidal-shaped cells in two days after treatment (Fig. 1B). By the fifth day, cells developed numerous lamellar processes and became spindle-shaped (Fig. 1C). The cytoplasmic compartments were more prominent: an increased number of mitochondria, polyribosomes, numerous vesicles and a growing number of multivesicular bodies (MVBs) could be observed in parallel with the inflammatory events of the surrounding tissue. A striking observation was the presence of thickened, electron-dense domains on the limiting membrane of MVBs with a fine coat of medium electron density on the cytosolic side (Fig. 1D). These specific membrane domains (referred from now on as MVB plaques) disappeared by the later stages of the MVB maturation process and could not be observed in the membrane of intermediate forms between MVB and lysosome (Fig. 1E).

**Figure 2:**
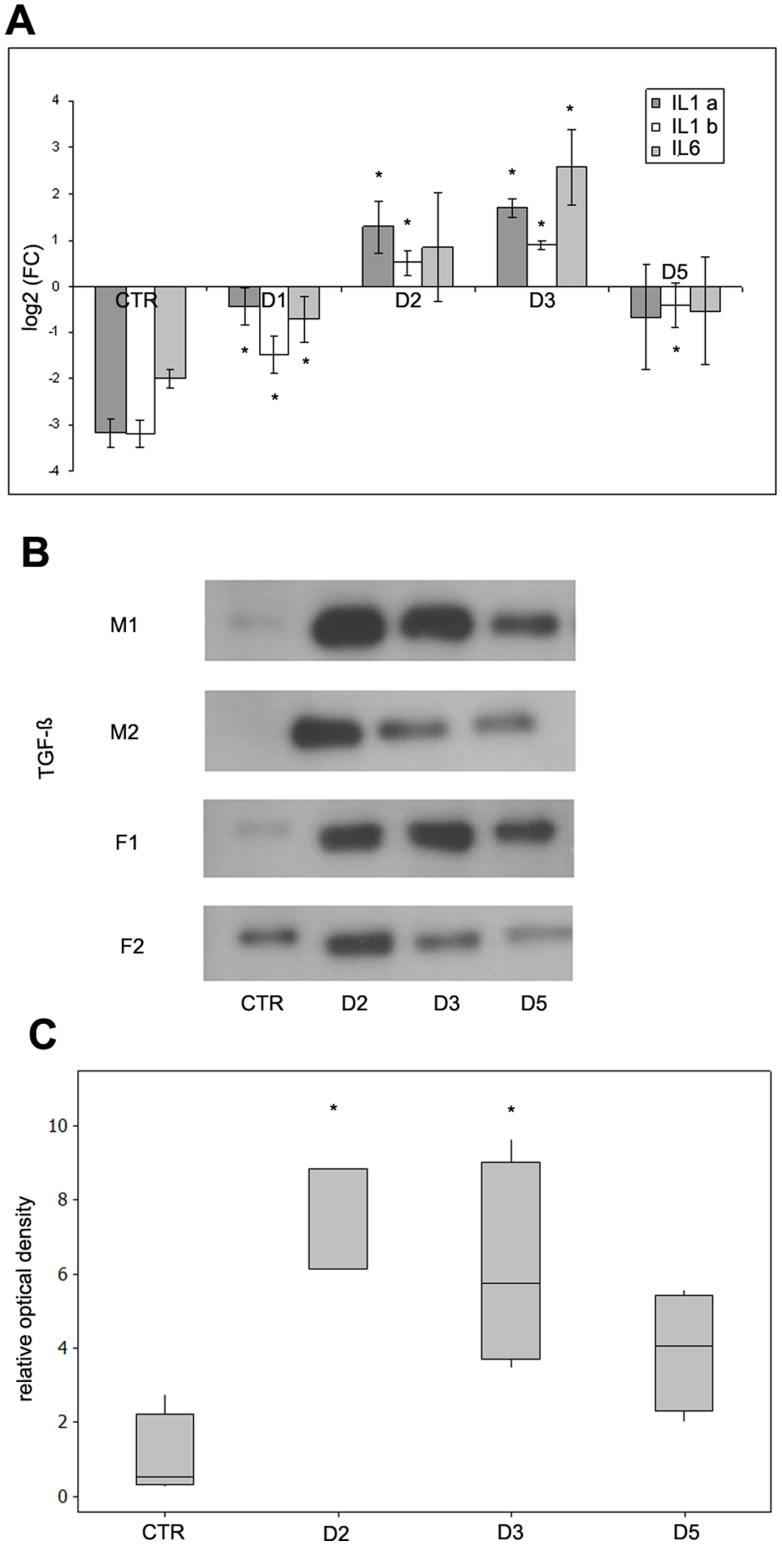
Expression levels of proinflammatory cytokines in mesothelial cells and peritoneal secretion of TGF-β in response to Freund's adjuvant treatment. (A) mRNA expression levels of IL-1alpha (IL1a), IL-1beta (IL1b) and IL-6 increase significantly until the third day where expression reaches a maximum, then declines to a similar level measured at the beginning of inflammation induction. (B) TGF-β in the peritoneal cavity follows the same pattern. Western blot using Pan-TGF-β antibody, 46 kDa. M: male F: female. (C) Relative levels of secreted TGF-β measured by densitometry. The asterisks show significant differences from the control group (p<0,05).

In addition to ultrastructural changes, it was of primary importance to see if Freund's adjuvant treatment induces inflammatory responses in our *in vivo* system at a molecular level as well. To verify this, we determined the expression levels of pro-inflammatory cytokines in mesothelial cells as they are well-known to be involved in immune responses and inflammatory processes. The results of quantitative RT-PCR showed that mRNA expression levels of interleukin type 1alpha and type 1beta and also interleukin 6 increased in mesothelial cells by Freund's adjuvant treatment (Fig. 2A). The elevated mRNA levels of these cytokines correlated with the time-scale of the observed morphological changes during the inflammatory events: expression levels of mRNAs had a peak on D3 followed by a significant downregulation that could be observed from the fifth day indicating the termination of the inflammatory response. Our Western blot data showed that TGF-β was secreted into the peritoneal cavity (Fig. 2B, C) and the secretion of the cytokine was in accordance with the morphological changes and with the expression levels of inflammatory cytokines indicating its role in EMT.

### Estrogen receptor alpha (ER-α) is expressed in mesothelial cells

**Figure 3:**
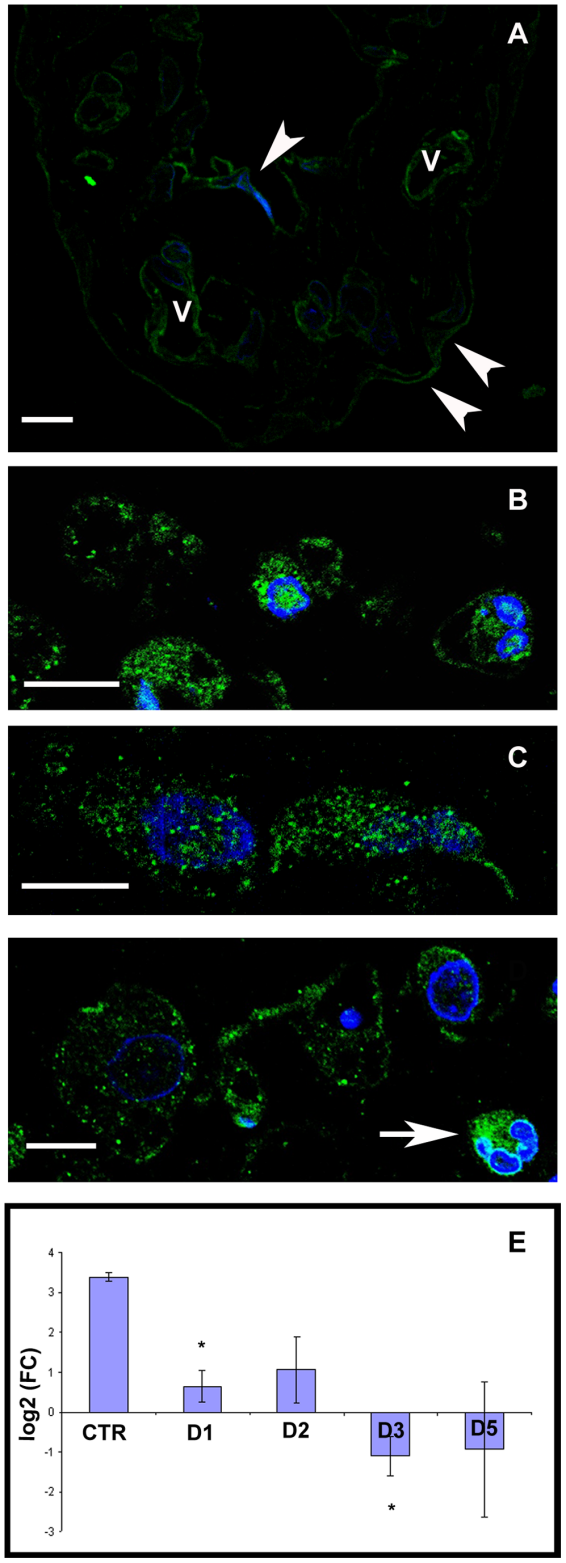
ER-α immunolabeling on control and Freund's adjuvant-treated mesothelial cells. Confocal micrographs of semithin frozen sections (A) Mesothelial cells are present on both sides of the connective tissue (white arrowheads). The labeling lines out the contour of mesothelial cells and can be detected in the wall of blood vessels (V) as well. (B–D) Parallel with the rounding up of mesothelial cells the distribution of ER-α becomes better detectable: labeling can be observed in the nucleus, cytoplasm as well as on the plasma membrane both on the second (B) and third (C) days after treatment.(D) By the fifth day, many of the mesothelial cells express ER-α mostly along the plasma membrane observable as small punctate structures. The connective tissue contains different cell types among which granulocytes express ER-α extensively (white arrow). Nuclei are stained with DAPI (blue). Scale bars: 10 µm. (E) ER-α mRNA level measured in mesothelial cells is significantly downregulated in response to treatment compared to control group. (asterisks: p<0,05).

Since mesothelial cells can serve as one of the sources of activated macrophages upon Freund's adjuvant treatment and it is known that macrophages express ER-α, the question arose whether mesenteric mesothelial cells also express the molecule? Our present results show that ER-α is present in mesothelial cells both under steady state and inflammatory conditions. As control cells were flat and the cytoplasm formed a thin rim around the nucleus, it was difficult to define the exact nuclear, cytoplasmic or plasma membrane (PM) localization of the marker with light microscope (Fig. 3A). The morphological changes of the cells upon inflammation enabled us to better detect the localization of ER-α. Confocal microscopical results after immunofluorescence detection of ER-α showed that labeling appeared in the nucleus, cytoplasm as well as in the plasma membrane and on the consecutive days after treatment the intensity of the labeling increased (Fig. 3B–D). In those mesothelial cells that had entirely lost the connection with the basal lamina, significant ER-α labeling was found on the plasma membrane and this distribution was clearly apparent on D5 samples (Fig. 3D). Distribution of labeling along the PM had been changed during the inflammatory process: while in control samples the labeling showed a continuous line at the PM (Fig. 3A), it clustered at certain areas of the plasma membrane in treated cells.

Since we found no detectable differences in ER-α labeling between female and male animals, we further carried out our experiments with samples taken from male rats if not indicated otherwise.

To further clarify the presence of ER-α in mesothelial cells we measured its expression at the mRNA level by quantitative RT-PCR. Our results revealed that control mesothelial cells expressed ER-α mRNA and it showed a significant downregulation during the progression of the inflammatory process (Fig. 3E). (We found the same pattern of changes in the mRNA expression levels of ER-β and G protein-coupled receptor 30, GPR30 as well (Fig. S1.).)

### Estrogen receptor alpha associates with caveolin-1 both in non-treated and treated mesothelial cells

**Figure 4:**
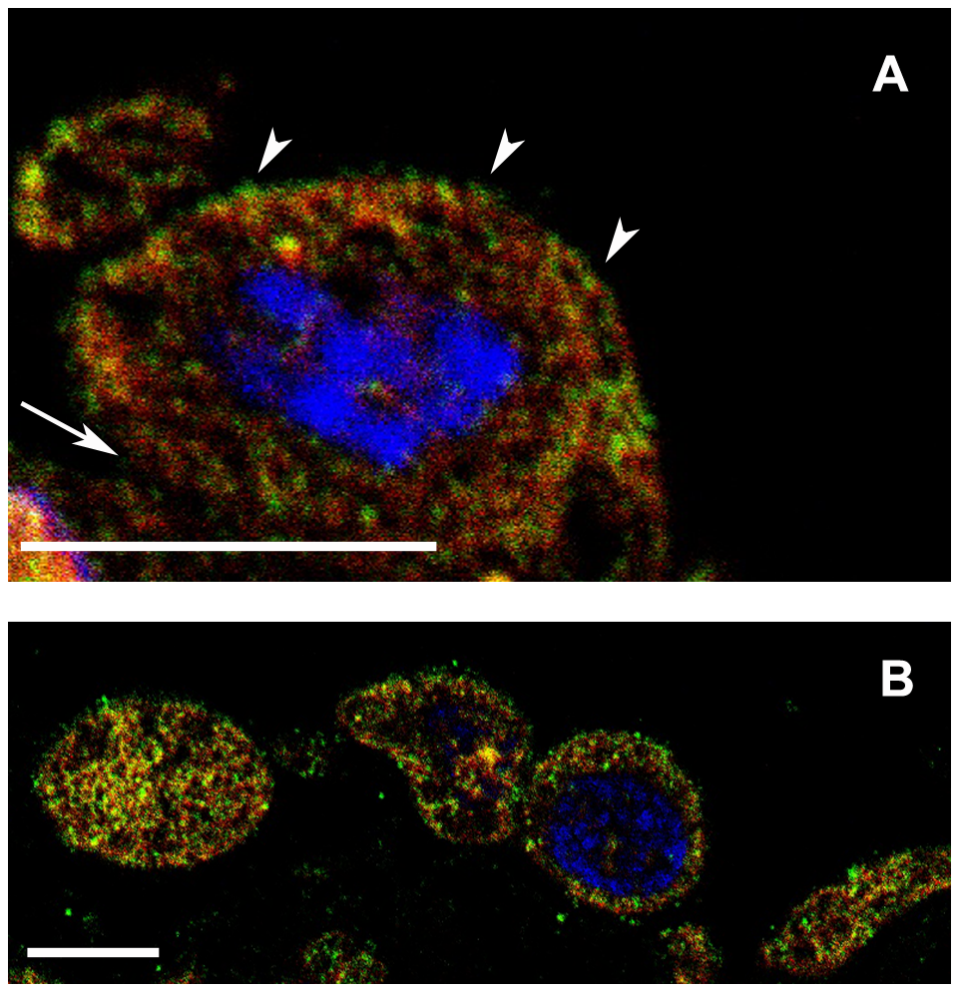
ER-α associates with caveolin-1 and its distribution changes upon inflammatory stimuli. Semithin frozen sections labeled with antibodies against ER-α (green) and caveolin-1 (red). Strong colocalization (orange) can be observed. (A) Two days after treatment, mesothelial cells (still attached to the basal lamina) show abundant expression of ER-α which is colocalized with caveolin-1 inside the cytoplasm and on the luminal side (arrowheads) of the cells. Less immunolabeled structures are present on the opposite side of the cell (arrow). (B) In the consecutive day (D3) when cells become apolar and lose contact with the basal lamina, a more intensive co-labeling can be observed over the plasma membrane as well as inside the cytoplasm with no asymmetric distribution. Nuclei are stained with DAPI (blue) Scale bars: 10 µm.

**Figure 5:**
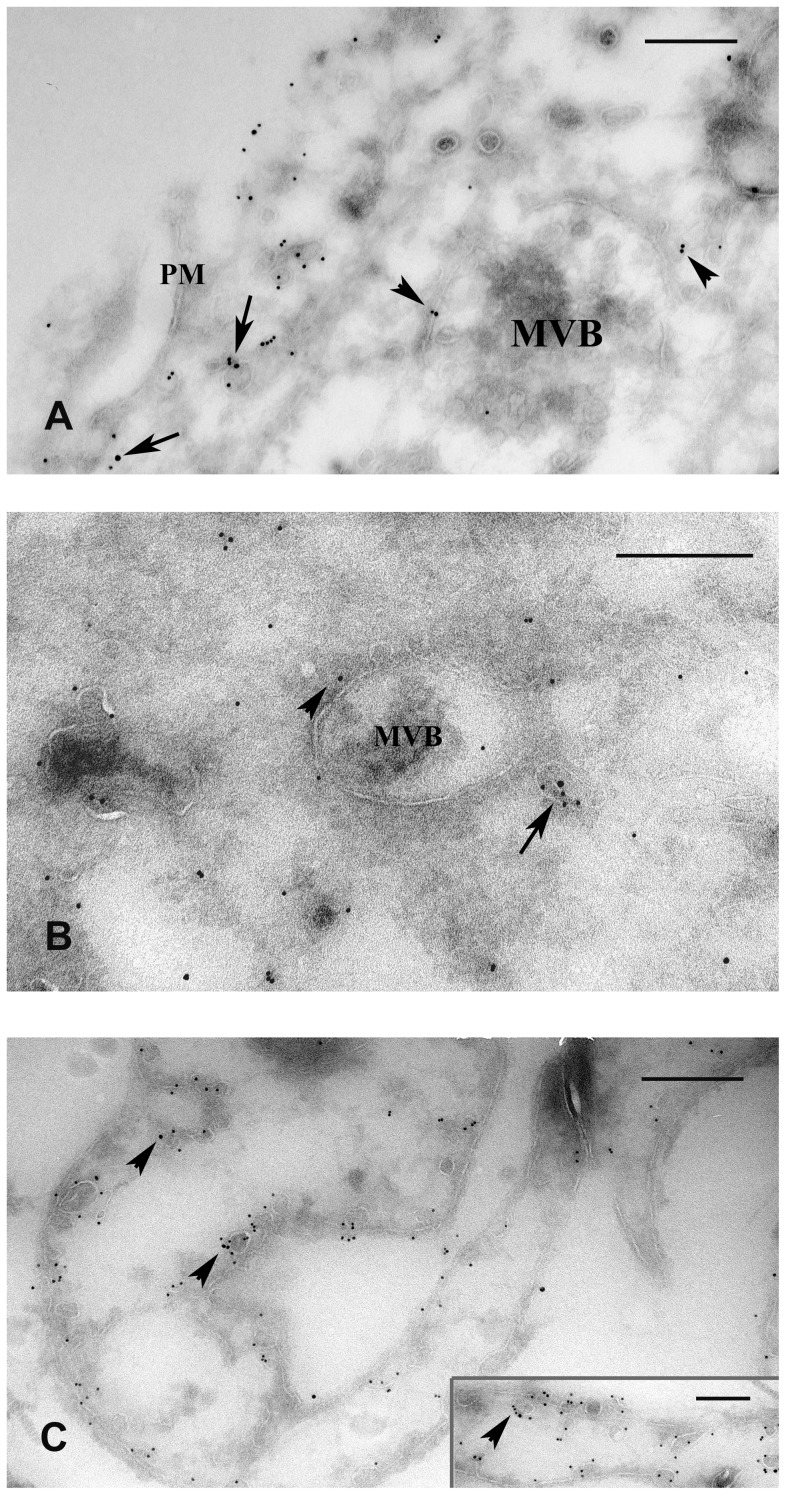
Electron microscopic immunolabeling on ultrathin frozen sections to show ER-α distribution in caveolin-positive lipid rafts and/or caveolae. ER-α was labeled with 15-nm gold particles and caveolin-1 with 10-nm gold. (A) ER-α can be found both in the plasma membrane (PM) associated with caveolin-1 (arrows) and (B) deeper inside the cytoplasm (arrow) in caveolae upon inflammation. There were no significant differences in the distribution of ER-α between D3 (A) and D5 (B). (A, B) During multivesicular body (MVB) formation, ER-α was found in the limiting membrane of these organelles and in caveolae in their close environment (arrowheads). (C) In non-treated cells ER-α occured in caveolin positive omega-shaped invaginations of the PM. This pattern of the markers was detectable both on the luminal and the basolateral surfaces (arrowheads) in control mesothelial cells. The bars represent (A) 250 nm, (B) 400 nm (C) 333 nm, insert 250 nm.

Since there are controversial data in the literature on the subcellular localization of ER-α in various cell types, we carried out double immunolabeling to detect the fine localization of ER-α as well as their possible co-localization with the coat protein caveolin-1. Our confocal microscopical results show that under inflammatory conditions ER-α colocalized with caveolin-1 both inside the cytoplasm and in the PM (Fig. 4). There were no differences in the distribution of the two markers between D3 and D5 (data not shown). However, in those mesothelial cells that were partially attached to the basal membrane, there was a remarkable colocalization of ER-α and caveolin-1 on the luminal surface of the cells (Fig. 4A). In contrast, with the disintegration of the basal membrane from the third day, ER-α appeared all over the plasma membrane in caveolin-positive membrane domains and colocalization could be detected also inside the cytoplasm (Fig. 4B). To obtain a more precise view on the localization of ER-α at the ultrastructural level, we carried out immunocytochemistry on ultrathin frozen sections of both non-treated and treated cells. Consistent with the immunofluorescence data, the same distribution of ER-α and caveolin-1 could be found on double-labeled ultrathin frozen sections of treated cells. The immunoelectron microscopic results clearly showed that at the peak time of inflammation (D3 and D5) ER-α occurred not only at the plasma membrane but appeared also inside the cytoplasm (Fig. 5A, B). Here ER-α (together with caveolin-1) was localized in forming or mature MVBs either in their limiting membrane, in association with caveolin-1 or in caveolae, in close vicinity of MVBs (Fig. 5A, B). In untreated, control mesothelial cells ER-α labeling was accumulated along the plasma membrane on both the luminal and basolateral sides of the cells, preferentially in caveolin-positive vesicles, caveolae (Fig. 5C). ER-α labeling was also observed inside the nuclei of both untreated and treated mesothelial cells, but to a lesser extent.

## Discussion

Epithelial-mesenchymal transition (EMT) is a complex mechanism that is characterized by a series of events including loss of cell-cell junctions and cell-matrix adhesion, reorganization of cytoskeleton resulting in loss of apical-basolateral polarity and assuming a mesenchymal chararacter such as spindle-shape morphology and motility. Due to its role in tumor metastasis as well as in normal embryogenesis and wound healing, the EMT is currently under intensive investigations [1]]–[[5].

In a previous paper we have shown that rat mesenteric mesothelial cells undergo EMT upon inflammatory stimuli induced by Freund's adjuvant treatment [26]. In the present paper we furnish additional evidence for the inflammatory response of the tissue by showing that the morphological changes at the ultrastructural level were in correlation with the expression levels of pro-inflammatory cytokines (IL 1α, 1β and IL-6) measured in mesothelial cells. This finding further supports the theory that mesothelial cells may contribute to the amplification of inflammatory responses by secreting interleukines and in this way, they are able to play an active role in peritoneal inflammatory events [34]]–[[36]. Proinflammatory cytokines (TNFα, IL1, IL6) are well known to induce immune and inflammatory responses by binding to their receptors. The major source of proinflammatory cytokines is the macrophages that produce plethora of these molecules including TNFα, IL-1, IL-6, Il-12, IL-15 and IL-18 [37]. Macrophages also secrete TGF-β at the site of injury that has a role in inducing EMT [1], [2], [5]. TGF-β is an important morphogenic factor initiating and maintaining EMT via different signaling routes (Smad-dependent, Smad-independent). Smad-independent signaling pathways including the activation of Erk, JNK, p38 MAP kinase cascades are considered to help and complete the process of TGF-β induced EMT. Although there are limited data about the exact mechanisms and biological consequences of these accessory signaling routes, it is apparent that TGF-β induced canonical and non-canonical pathways intersect and both are essential for effective signaling [38]]–[[41]. Similarly, we found significant elevation in the secreted TGF-β concentration as the inflammation progressed indicating its main role to organize the EMT upon treatment in our *in vivo* system as well.

Earlier results from our laboratory have shown that upon adjuvant stimulation mesothelial cells are detached from the basal lamina and assume a macrophage character expressing ED1, a macrophage marker [27] and they can serve as a source of activated macrophages during inflammatory events [28]. Since macrophages are known to express ER-α [29], [30], the question arose whether treated and untreated mesothelial cells do produce ER-α as well? Our present morphological (confocal and immuno-EM) and biochemical (qRT-PCR) results show that ER-α is really present in treated as well as in control mesothelial cells. This finding is of special interest since recently it was shown that in addition to its well known genomic role as a transcription factor, ER-α has several non-genomic functions. Nowadays it became generally accepted that a certain amount of ER-α resides in the plasma membrane and is thought to be responsible for inducing both genomic and non-genomic actions by activating different cascades, like PKC, Src kinase, MAPK and PI3K [17]]–[[23]. Our present light microscopic data showed that in parallel with the inflammatory events the labeling intensity of ER-α was significantly increased and could be detected in the cytoplasm, plasma membrane as well as in the nucleus. Besides the novel finding that ER-α is expressed in mesenteric mesothelial cells, the nuclear pool of the receptor was less pronounced and unchanged during the inflammatory events. This directed our focus to study further the cytoplasmic/plasma membrane pool of ER-α. In mesothelial cells, still in contact with the basal lamina, the distribution of plasma membrane ER-α pool showed a certain polarity with a predominant localization on the luminal side, while after being detached ER-α was distributed all over the plasma membrane. This redistribution of ER-α in the plasma membrane indicates loss of cell polarity and is a further proof for the transformation of a polarized epithelial cell into a non-polarized mesenchymal cell.

An interesting finding in our study was that the mRNA level of ER-α showed an inverse correlation with the labeling intensity of the protein on our immunocytochemical specimens demonstrating that on a transcriptional level ER-α was downregulated. Several studies have already described that mRNA and protein levels do not always correlate. The exact mechanism is still unclear, a possible explanation for this phenomenon can be a protein-protein interaction, the altered turnover of the molecule and posttranscriptional regulation by microRNAs [42]]–[[49].

A further finding in this study is that both plasma membrane ER-α and the cytosolic receptor pool were predominantly associated with caveolin-1 protein. Similar findings are known from literature showing that ER-α resides in caveolin positive raft compartments of the plasma membrane in many cells [14], [50]. Caveolin-1 is the major protein component of special highly hydrophobic membrane domains, called caveolae. Caveolae are omega-shaped, small plasma membrane invaginations that are found in most mammalian cell types. They play important role in many cellular functions including vesicular transport, endocytosis, transcytosis and are also described to be involved in different signal transduction events [51]]–[[55]. Collectively these data suggest that caveolae are platforms of plasma membrane where, besides other transduction proteins, ER-α is also present. The fine structural distribution of the receptor demonstrated by our immuno-electron micrographs suggests that plasma membrane ER-α is internalized upon inflammatory stimuli and is targeted to the classical endosomal pathway: towards cytoplasmic organelles, multivesicular bodies. This finding is not unusual, since endocytosis of membrane receptors and their transport along the endosomal pathway have been described in other signaling systems as well [56], [57]. Since caveolae are often considered as ‘signaling platforms’ [58], [59], caveola-mediated endocytosis results in a) removing signaling molecules (ERalpha, src) from the cell surface b) providing pathway for biologically important signal transducers to reach the proper cytoplasmic platforms (late endosomes, MVBs) where the microenvironment is optimal for their interaction. Considering the colocalization of ER-α with cavolin-1, it is likely that internalization is caveola-mediated. This suggestion is further supported by Christensen et al. showing that inhibition of Cav-1 by siRNA reduced membrane estrogen-receptor α levels [60]. Our further observation was that by D6 the plasma membrane of mesothelial cells devoids both clathrin-coated vesicles and caveolae (Fig. S2) indicating that internalization of signaling molecules (ER-α, Src-data not shown) in caveolae is presumably important in the process of EMT.

It is known that the association of caveolin and ER-α can activate MAP kinase pathways by interacting either with the signaling proteins or with each other [61]]–[[62]. The best studied interaction in raft domains of the plasma membrane is the induction of Erk pathway through Src/Shc/Grb2/Sos protein complex formation where these proteins physically interact with both caveolin-1 and ER-α and are activated after TGF-β stimulus [64],[65]. Taking into consideration these data, we suppose that ER-α might have a role in our system by modifying the activity of main signaling pathways via maintaining physical interaction with signaling proteins such as caveolin-1 and Src (data not shown).

There are limited data available about the localization of the elements of MAPK cascades inside the cytoplasm. The Erk pathway is well characterized and these studies illustrate the role of endosomal compartments. Either the upstream elements of these non-canonical pathways are described to occur in the plasma membrane/membrane of early endosomes or the downstream signaling elements are bound to late endosome/MVB membranes via different adaptor proteins [66]]–[[68]. One of these adaptor proteins (p18) has already been proved to associate with cavolin -1 indicating that upon internalization, the lipid compound of caveolae - that partially form the limiting membrane of these endosomal compartments- have importance [69]. They presumably maintain a physical platform to bind the elements of signaling proteins and in this way, they can orchestrate the spatial and temporal regulation of different signaling routes. In this paper we showed that ER-α was found either in the limiting membrane of MVBs colocalizing with caveolin-1 or in caveolae in close vicinity to late endosomal/MVB compartments. Comparing the distribution of the receptor and our EM images showing the MVB plaques, we hypothesize that the membrane of endosomal compartments/MVBs can form platforms for molecular clusters and can organize spatial and temporal distribution of signaling proteins. Although our results do not prove directly whether PM ER-α induces signaling from the plasma membrane and whether it controls accessory pathways, however, its association with caveolin-1 and Src (data not shown) strongly suggest that it might play a role in signaling events.

Recent investigations also revealed that ER-α can enhance the degradation of Smad proteins inside the nucleus via ubiquitin-proteasome system and inhibit TGF-β signaling [24]. These data illustrate the role of ER-α as a non-genomic nuclear modifier of TGF-β signaling cascade.

The main aim of this study was to bring into focus the role of *in vivo* cell populations that can help examine the biological processes of EMT. Our work also draws attention to the role of cellular compartmentalization (caveolae and endosomes/MVBs) that can maintain platforms for possible interactions of regulatory proteins and spatially-temporally might orchestrate the signaling events. Our morphological observations and biochemical data can contribute to form a potential model whereby possible ERα-TGFβ cross-talk can further be investigated *in vivo*. However fine details of the mechanisms and further functional evaluations are essential to clarify the exact role of ER-α in mesothelial cells under inflammatory conditions.

## Supporting Information

Figure S1 **The changes of mRNA expression levels of ER-β and GPR30 upon inflammatory stimuli.** Similarly to ER-α, the mRNA levels of ER-β and G protein-coupled receptor 30, GPR30 showed a significant downregulation upon treatment compared to control group. (asterisks: p<0,05).
(TIF)

Figure S2 **Electron micrograph of a mesothelial cell six days after treatment**. The plasma membrane of mesothelial cells devoid both clathrin-coated vesicles and caveolae six days after inducing inflammation. Observe the ‘emptiness’ of the cell surface of the mesothelial cell. N: nucleus. Bar represents 833 nm.
(TIF)
