# Supplementary figures and images for: Estrogen Receptor Alpha Is Expressed in Mesenteric Mesothelial Cells and Is Internalized in Caveolae upon Freund's Adjuvant Treatment
Source: PLoS One. 2013 Nov 14;8(11):e79508. doi: 10.1371/journal.pone.0079508 (PMC3828353; doi:10.1371/journal.pone.0079508)

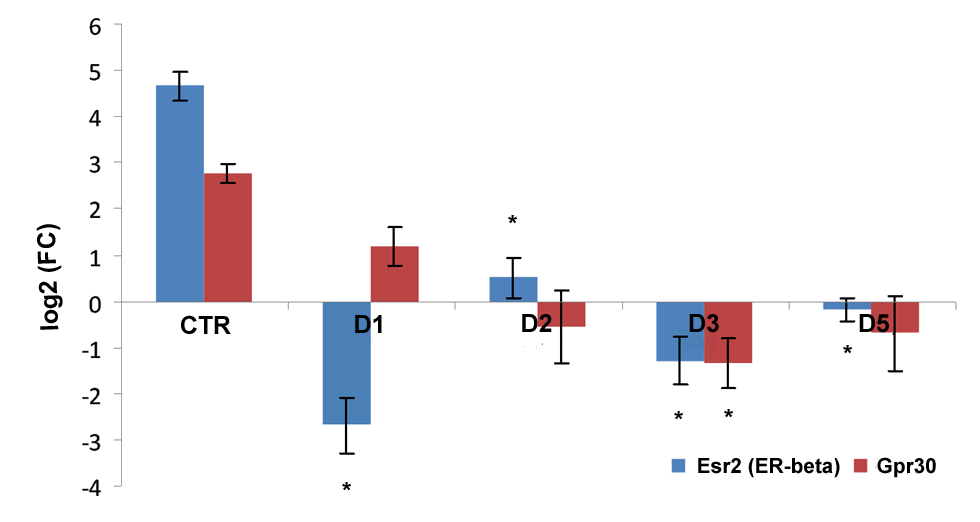

Supplement: Figure S1 — The changes of mRNA expression levels of ER-β and GPR30 upon inflammatory stimuli. Similarly to ER-α, the mRNA levels of ER-β and G protein-coupled receptor 30, GPR30 showed a significant downregulation upon treatment compared to control group. (asterisks: p<0,05). (TIF) [file pone.0079508.s001.tif]

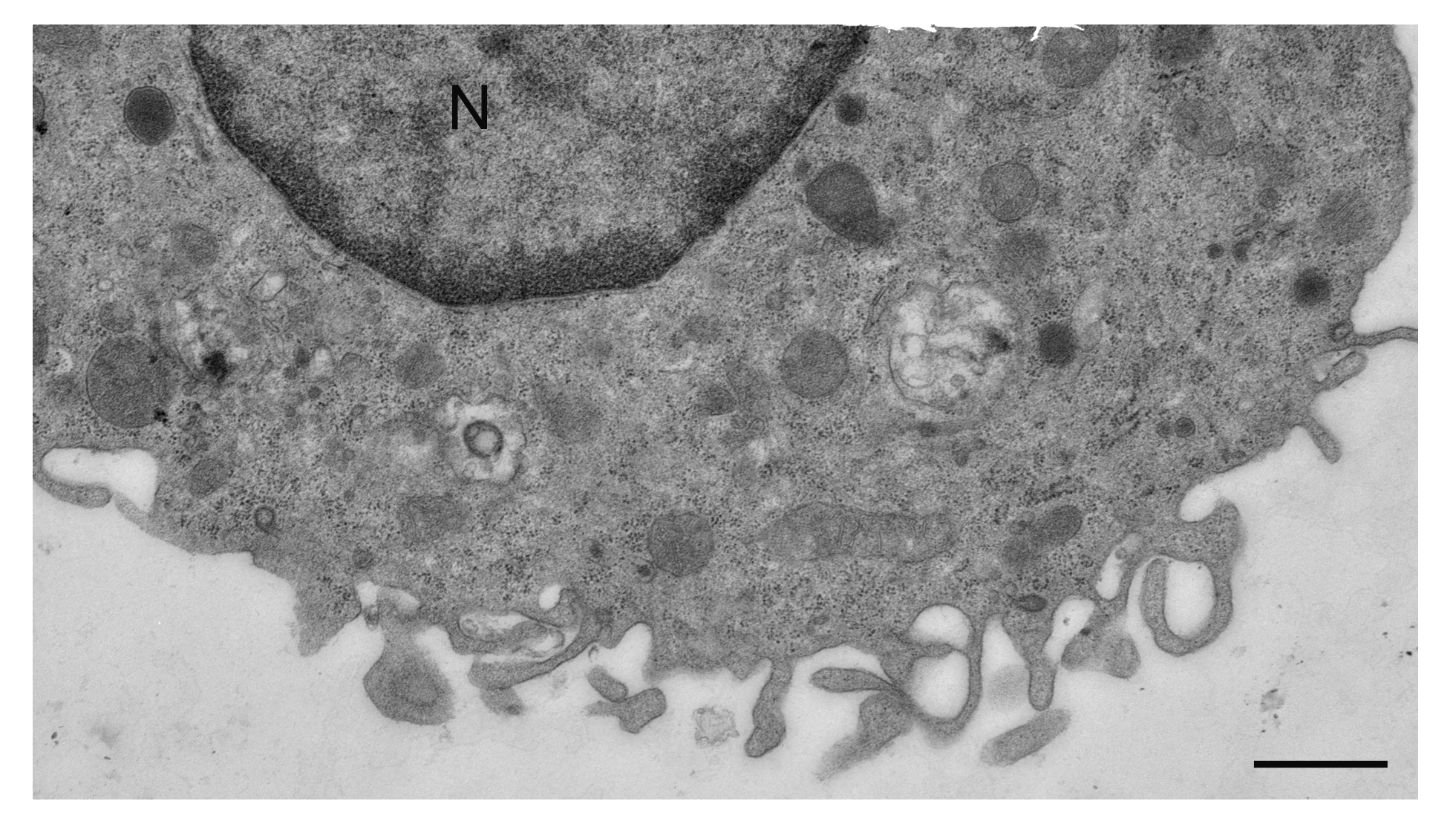

Supplement: Figure S2 — Electron micrograph of a mesothelial cell six days after treatment. The plasma membrane of mesothelial cells devoid both clathrin-coated vesicles and caveolae six days after inducing inflammation. Observe the ‘emptiness’ of the cell surface of the mesothelial cell. N: nucleus. Bar represents 833 nm. (TIF) [file pone.0079508.s002.tif]
